# A Modified Cell-Penetrating Peptide Enhances Insulin and Oxytocin Delivery across an RPMI 2650 Nasal Epithelial Cell Barrier In Vitro

**DOI:** 10.3390/pharmaceutics16101267

**Published:** 2024-09-28

**Authors:** Sara Wong, Alexander D. Brown, Abigail B. Abrahams, An Nisaa Nurzak, Hoda M. Eltaher, David A. Sykes, Dmitry B. Veprintsev, Kevin C. F. Fone, James E. Dixon, Madeleine V. King

**Affiliations:** 1 Division of Physiology Pharmacology and Neuroscience, School of Life Sciences, University of Nottingham Medical School, Queen’s Medical Centre, Nottingham NG7 2UH, UK; sara.wong@imperial.ac.uk (S.W.); dominickearney0@gmail.com (A.D.B.); a.abrahamswilliams@gmail.com (A.B.A.); david.sykes@nottingham.ac.uk (D.A.S.); dmitry.veprintsev@nottingham.ac.uk (D.B.V.); kevin.fone@nottingham.ac.uk (K.C.F.F.); 2 Regenerative Medicine and Cellular Therapies, School of Pharmacy, Biodiscovery Institute (BDI), University of Nottingham, University Park Campus, Nottingham NG7 2RD, UK; annisaa.nurzak@nottingham.ac.uk (A.N.N.); hoda.eltaher@nottingham.ac.uk (H.M.E.); james.dixon@nottingham.ac.uk (J.E.D.)

**Keywords:** insulin, oxytocin, cell-penetrating peptide, glycosaminoglycan-GAG-binding enhanced transduction, RPMI 2650, transcytosis, transepithelial delivery, nasal drug delivery

## Abstract

**Background/Objectives:** Peptide-based treatments represent an expanding area and require innovative approaches to enhance bioavailability. Combination with cell-penetrating peptides (CPPs) is an attractive strategy to improve non-invasive delivery across nasal epithelial barriers for systemic and direct nose-to-brain transport. We previously developed a modified CPP system termed Glycosaminoglycan-binding Enhanced Transduction (GET) that improves insulin delivery across gastrointestinal epithelium. It contains a membrane docking sequence to promote cellular interactions (P21), a cationic polyarginine domain to stimulate uptake (8R) and an endosomal escaping sequence to maximize availability for onward distribution (LK15). It is synthesized as a single 44-residue peptide (P21-LK15-8R; PLR). Methods: The current research used in vitro assays for a novel exploration of PLR’s ability to improve the transport of two contrasting peptides, insulin (51 residues, net negative charge) and oxytocin (9 residues, weak positive charge) across an RPMI 2650 human nasal epithelial cell barrier cultured at the air–liquid interface. Results: PLR enhanced insulin transcytosis over a 6 h period by 7.8-fold when used at a 2:1 molar ratio of insulin/PLR (*p* < 0.0001 versus insulin alone). Enhanced oxytocin transcytosis (5-fold) occurred with a 1:10 ratio of oytocin/PLR (*p* < 0.01). Importantly, these were independent of any impact on transepithelial electrical resistance (TEER) or cell viability (*p* > 0.05). Conclusions: We advocate the continued evaluation of insulin–PLR and oxytocin–PLR formulations, including longer-term assessments of ciliotoxicity and cytotoxicity in vitro followed by in vivo assessments of systemic and nose-to-brain delivery.

## 1. Introduction

Peptide-based treatments are a growing sector of the pharmaceutical market, with over 80 approved and a further 200 in clinical development [1]. They are more selective and potent than most small molecules because their size and structural complexity allow for better interaction with biological targets. However, they usually have poor oral bioavailability (<2%), so strategies to improve non-invasive delivery across oral, nasal and pulmonary epithelial barriers are very attractive. There is an array of options, including tight junction modulators that reduce barrier integrity, bio-adhesive systems that prolong the residence time and delivery devices to enhance access to the desired uptake site, as well as cell-penetrating peptides (CPPs) that increase cellular uptake. Numerous CPPs have been described, including cationic, amphipathic or hydrophobic sequences that bind covalently or non-covalently to their therapeutic cargoes and enhance barrier penetration via membrane translocation or endocytosis [2,3,4]. 

**Figure 1:**
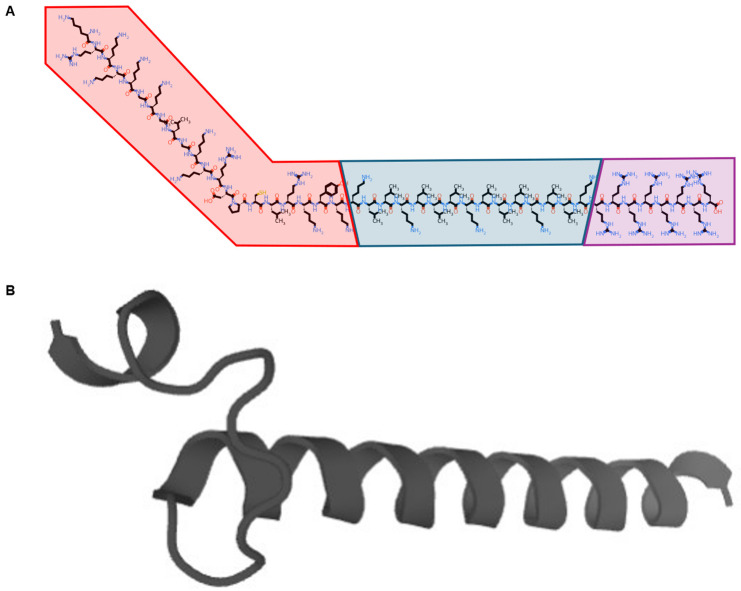
The amino acid sequence of the PLR peptide is KRKKKGKGLGKKRDPCLRKYKKLLKLLLKLLLKLLKRRRRRRRR. The images show (**A**) a simplified molecular-input line-entry system (SMILES) notation, with the heparan-binding domain from heparin-binding epidermal growth factor (P21, red), endosomal escaping sequence (LK15, blue) and cationic polyarginine CPP (8R, purple; produced using the PepSMI tool by NovoPro, https://www.novoprolabs.com/tools/convert-peptide-to-smiles-string, accessed on 22 September 2024) and (**B**) a two-dimensional model (produced using the PEP-FOLD3 tool [11] https://bioserv.rpbs.univ-paris-diderot.fr/services/PEP-FOLD3/, accessed on 22 September 2024).

Our group developed a modified CPP system termed Glycosaminoglycan-binding Enhanced Transduction (GET). It contains a membrane docking sequence that binds to cell surface heparan sulfate proteoglycans (a 21-residue heparan-binding domain from heparin-binding epidermal growth factor; P21) plus a cationic polyarginine CPP (8R) to stimulate uptake. Their combination has a synergistic effect on the internalization of cargo peptides via lipid raft-dependent endocytosis. It also contains an endosomal escaping sequence (LK15) to maximize cargo availability for onward distribution and is synthesized as a single 44-residue peptide (P21-LK15-8R; PLR: Figure 1) [5]. We recently showed that PLR combines with insulin to form a non-toxic nanocomplex, which enhances insulin transport across intestinal epithelium in vitro and can be orally administered to normalize blood glucose in diabetic mice [6]. The GET system has also been used to facilitate gene transfer to the lungs [7] and enhance vaccine uptake [8] and is being explored for regenerative medicine applications [9,10]. However, it has yet to be considered for nasal delivery.

Strategies to enhance nasal delivery are attractive for both systemic and direct nose-to-brain transport of therapeutic peptides [12]. For example, there is interest in improving nasal insulin bioavailability to manage blood glucose levels [13,14] because the required doses are currently approximately 20 times higher than those for injection, and glycemic control remains inadequate [15] (despite the respiratory region of the nasal cavity having a large surface area and extensive vasculature). Furthermore, the central actions of insulin are potentially desirable in Alzheimer’s disease, traumatic brain injury and cerebral ischemia [16]. Although application to the olfactory epithelium enables transport to the brain (via olfactory and trigeminal nerve pathways that bypass the blood–brain barrier), it is recognized that combination with CPPs can improve delivery to relevant brain regions [17]. Enhanced nose-to-brain delivery of numerous other peptides is also desirable for conditions like depression, Parkinson’s disease and Post-Traumatic Stress Disorder, with oxytocin receiving particular attention for Autism Spectrum Disorders and schizophrenia [12]. 

The current work used a series of in vitro assays to explore the potential of PLR to improve the nasal delivery of two contrasting peptides, insulin (51 residues, net negative charge) and oxytocin (9 residues, weak positive charge). We used the RPMI 2650 human nasal epithelial cell line, which has been extensively characterized and is particularly suitable for predicting nasal delivery [18,19,20,21,22,23]. We began with flow cytometry and confocal microscopy to examine the impact of PLR on the uptake of fluorescent-labeled insulin (fluorescein isothiocyanate-insulin; FITC-insulin), as with our previous work using gastrointestinal epithelium [6], and then progressed to the assessment of FITC-insulin transcytosis across a nasal epithelial barrier cultured at the air–liquid interface. We also examined the effect of insulin–PLR nanocomplexes on RPMI 2650 barrier integrity (transepithelial electrical resistance; TEER) and viability (PrestoBlue^TM^ assay [24]). Having obtained encouraging findings, we adopted similar approaches with oxytocin–PLR. Because the sequence of oxytocin itself is not amenable to fluorescent labeling [25], we developed a calcium fluorimetry reporter assay as an alternative readout for oxytocin levels in media taken from either side of our RPMI 2560 barrier. Calcium fluorimetry detects the rapid rise in intracellular calcium levels that follows the activation of Gα_q_-coupled oxytocin receptors and the intracellular release of inositol triphosphate. We chose the oxytocin receptor-expressing breast cancer cell line Hs 578T [26] for this reporter assay over alternatives like the MCF-7 line [27] because Hs 578T cells show a more robust concentration-dependent calcium response to oxytocin [28]. We were able to identify both insulin–PLR and oxytocin–PLR concentration ratios that enhanced transcytosis across an RPMI 2650 nasal epithelial cell layer without reducing barrier integrity or cell viability. These findings are encouraging given that the transport of other peptides and peptide-like molecules across olfactory or respiratory epithelium is lower than that across gastrointestinal epithelial barriers [29,30], and they are relevant to our previous work with insulin–PLR [6]. 

## 2. Materials and Methods

### 2.1. Peptides

Recombinant human insulin sodium salt (91077C, 27.5 IU/mg), FITC-insulin (I3661) and heparin (H3149) were obtained from Sigma-Aldrich, Gillingham, UK (purity > 98%), and oxytocin acetate was purchased from Bachem, Saint Helens, UK (4016373, purity > 98%). PLR was designed by our group [5] and PLR, FITC-PLR and tetramethylrhodamine isothiocyanate-PLR (TRITC-PLR) peptides were custom-synthesized by Synpeptide (Shanghai, China) upon request (purity > 95%). 

### 2.2. Cell Lines

RPMI 2650 human nasal epithelial cells (CCL-30; ATCC, Manassas, VA, USA) for uptake, transcytosis and viability studies were cultured in Eagle’s Minimum Essential Medium (EMEM, ATCC) supplemented with 10% heat-inactivated Fetal Calf Serum (FCS, Sigma-Aldrich), 1% penicillin/streptomycin (Invitrogen, Oxford, UK) and 1% glutamine (Sigma-Aldrich). Hs 578T breast cancer cells (University of Nottingham’s Cancer Research Nottingham NCI-60 cell line facility) for our calcium fluorimetry reporter assay were cultured in Dulbecco’s Modified Eagle’s Medium (DMEM, Sigma-Aldrich) with 10% heat-inactivated FCS containing 0.1% bovine insulin (Sigma-Aldrich). These cells were chosen because they express functional oxytocin receptors [26,28] and were used purely to readout oxytocin levels in culture media from either side of the RPMI 2650 barrier. We do not intend to imply any future role for insulin–PLR or oxytocin–PLR in oncology. All cultures were maintained in T75 flasks (Nunc^TM^; Thermo Fisher Scientific, Loughborough, UK) in a humidified incubator (Heracell; Thermo Fisher Scientific) at 37 °C with 5% CO_2_ and split with 0.25% trypsin EDTA (Sigma-Aldrich) when 70–90% confluence was attained.

### 2.3. Insulin–PLR and Oxytocin–PLR Nanocomplex Formation

We showed that insulin readily complexes with PLR through electrostatic interaction to form particles with an average diameter of 140 nm and a highly positively charged surface potential of +28.16 mV [6]. FITC-insulin–PLR or insulin–PLR nanocomplexes for the current study were formed using our previously described method, where insulin and PLR stock solutions were mixed in serum-free culture medium in polypropylene tubes and incubated at room temperature for 15 min to allow nanocomplex formation. Mixtures were used immediately after the 15 min complexing period and resulted in a final applied concentration of 3.4 μM of insulin with 0.017–68 μM PLR (corresponding to molar insulin/PLR ratios ranging from 200:1 to 1:20). We used the same approach for oxytocin–PLR nanocomplexes and applied final concentrations of 0.1 μM oxytocin and 0.001–10 μM PLR or 1.2 μM oxytocin and 0.012–120 μM PLR (corresponding to molar oxytocin/PLR ratios ranging from 100:1 to 1:100). As this study represents the first combination of oxytocin with PLR, we verified complex formation using a similar FITC-PLR and TRITC-PLR quenching assay to our early work with insulin–PLR [6] (Section 2.7.1) as well as additional bioactivity studies in our Hs 578T reporter assay (Section 2.7.2), which suggested a non-covalent interaction. We also used dynamic light scattering (DLS) and transmission electron microscopy (TEM) to characterize oxytocin–PLR particles (Section 2.7.3). 

### 2.4. Effect of PLR on Insulin Uptake by RPMI 2650 Cells

#### 2.4.1. Flow Cytometry

RPMI 2650 cells were seeded in 12-well plates (Thermo Fisher Scientific; 200,000 cells in 1 mL of supplemented EMEM per well). The following day, media were aspirated, and wells were rinsed with 1 mL of phosphate-buffered saline (PBS; Sigma-Aldrich) and then filled with 450 μL media plus 50 μL nanocomplex solution. Initial concentration–response studies assessed our full range of five insulin/PLR ratios following a 24 h incubation. Subsequent time course studies from 1 to 6 h were restricted to the 20:1 combination because this represented the lowest PLR concentration to enhance 24 h uptake, and our aim here was to see if an effect could also be detected at earlier time points. At the end of the incubation period, cells were washed with 1 mL PBS and then harvested for flow cytometry using 500 μL 0.25% trypsin-EDTA, which was combined with 500 μL of media and centrifuged (300 G, 5 min). Cell pellets were resuspended in 500 μL of 4% paraformaldehyde (PFA: Sigma-Aldrich) and stored at 4 °C until analysis on a CtyoFlex S flow cytometer (Beckman Coulter, High Wycombe, UK; >20,000 events per sample) with FITC excitation at 488 nm and emission at 525 nm. Data were collected with Kaluza software (v2.1) and mean fluorescence intensity (MFI) after gating was used for statistical analysis.

#### 2.4.2. Confocal Microscopy

RPMI 2650 cells were seeded on glass cover slips designed to fit in 12-well plates (Thermo Fisher Scientific), and the following day, they were incubated with a 20:1 insulin/PLR ratio for 1, 4 and 6 h as described above. Cells were fixed (500 μL of 4% PFA, 10 min at room temperature), permeabilized (100 μL 0.1% Triton X-100; Sigma-Aldrich, 12 min at room temperature) and mounted (Fluoroshield mounting medium with DAPI; Sigma-Aldrich) with a PBS wash (500 μL) before each of these steps. Samples were viewed on a Zeiss LSM 800 confocal microscope (Zeiss, Cambourne, UK) with a 10× water immersion lens. DAPI and FITC were imaged with excitation at 405 nm and 488 nm, respectively, and emission at 426–491 nm and 505–585 nm, respectively. Frame sizes were set at 1024 × 1024, and laser power and gain were kept constant across samples.

### 2.5. Effect of PLR on Insulin Transcytosis across an RPMI 2650 Cell Barrier

RPMI 2650 cells were seeded on polystyrene capillary pore membrane inserts (12.07 mm diameter, 0.4 μm pore size; 66564; Greiner Bio-One, Stonehouse, UK) that fit into 12-well plates (250,000 cells in 500 μL of supplemented EMEM in the apical chamber with a further 1 mL of media in the basal chamber). After eight days, liquid-covered culture medium was removed from the apical chamber, and cells were cultured at the air–liquid interface for a further 12–13 days to stimulate the expression of physiologically relevant tight junction and drug transport proteins [18,19,22,31,32]. Medium was refreshed, and TEER was measured every two to four days using an EVOM2^TM^ epithelial voltohmeter (World Precision Instruments, Sarasota, FL, USA), with background conductance removed by subtracting acellular readings. TEER of human nasal mucosa is typically 60–180 Ω/cm^2^ [18], so only cultures with TEER above 60 Ω/cm^2^ were used for transcytosis or viability studies. 

To assess transcytosis, medium was aspirated, and cells were rinsed three times with 500 μL of PBS and then incubated in fresh serum-free media (500 μL apical and 1.5 mL basal) for 60 min to achieve equilibrium. Serum-free medium was used to limit the presence of additional glycosaminoglycans that would compete for the P21 domain of our delivery peptide [5]. Baseline TEER was measured, and then contents of the apical chamber were replaced with a combination of 450 μL serum-free media and 50 μL of prepared nanocomplex solution (at two molar concentration ratios, 20:1 and 2:1, shown to increase uptake without reducing epithelial cell viability under liquid covered culture). Separate cells were incubated with 4.4 kDa FITC-dextran which is a hydrophilic compound commonly used to assess paracellular transport [18] and will provide a conservative estimate of rates for insulin/PLR and oxytcin/PLR complexes (which are expected to be >2 kDa larger). At 1, 4 and 6 h, TEER measurements were repeated to determine peptide effects on barrier integrity, and then 100 μL samples of basal media was collected and stored (on ice and in the dark) while a corresponding volume of fresh serum-free media was replaced into the basal chamber. Collected samples were transferred to a black 96-well plate (Greiner Bio-One), and FITC-insulin or FITC-dextran concentrations were determined against a standard curve (0.017–3.4 μM) using a Tecan Infinite 200PRO plate reader (Tecan, Männedorf, Switzerland) with FITC excitation at 490 nm and emission at 520 nm. The apparent permeability coefficient (P_app_), which quantifies the rate at which molecules cross a membrane, was calculated according to the following Equation (1):

P_app_ = (dQ/dt)/(C_0_ × A), (1)

where dQ/dt = the flux of compound across the barrier (mmol/sec), C_0_ = the initial concentration of the compound in the apical chamber (mmol/cm^3^) and A = the surface area of the cell monolayer (cm^2^) [33].

### 2.6. Effect of Insulin–PLR Nanocomplexes on RPMI 2650 Cell Viability

To assess potential cytotoxic effects of insulin–PLR nanocomplexes on RPMI 2650 cells, we performed parallel viability assays alongside both uptake and transcytosis measurements. Cells were seeded and incubated as described above. After, the desired time nanocomplex solutions were replaced with PrestoBlue™ Cell Viability Reagent (Thermo Fisher Scientific) and diluted in a 1:10 ratio in Hanks’ Balanced Salt Solution (HBSS; Sigma-Aldrich). PrestoBlue™ reagent contains resazurin, which is converted by metabolically active viable cells to fluorescent resofurin [34]. After incubating for 60 min, fluorescence of 50 μL of samples was determined as described in Section 2.4, with excitation at 560 nm and emission at 590 nm. 

### 2.7. Demonstration of Oxytocin–PLR Nanocomplex Formation

#### 2.7.1. Fluorescence Quenching

To assess whether oxytocin would complex with PLR, we employed FITC-PLR and TRITC-PLR quenching assays. The principle is that fluorescent signals from each labeled molecule are reduced when interactions with neighboring molecules bring them into close proximity [35], i.e., when multiple PLR molecules combine with an oxytocin molecule, fluorescence should be reduced. FITC-PLR or TRITC-PLR PLR (20 μM) was incubated with increasing molar concentrations of oxytocin (0–400 μM, 0–20-fold molar excess), and fluorescence was measured after 15 min at room temperature, as described in Section 2.4, with TRITC excitation at 550 nm and emission at 580 nm. To evaluate the stability of the oxytocin–PLR interaction, we employed a dequenching assay. The principle is that signals from each labeled molecule increase when PLR decouples from oxytocin in response to heparin (0.001–100 μg/mL) competition [5] for the P21 domain.

#### 2.7.2. Bioactivity Studies in a Hs 578T Reporter Assay

Twenty-four hours prior to use, Hs 578T cells were seeded in black 96-well plates (80,000 cells in 100 μL of supplemented DMEM per well). On the test day, changes in intracellular calcium ([Ca^2+^]_i_) were measured using a Fluo-4 no wash calcium assay kit (F36206; Thermo Fisher Scientific). Briefly, medium was aspirated and replaced with 100 μL of 1× dye loading solution (Fluo-4 NW mix in 1× HBSS) containing 20 mM 4-(2-hydroxyethyl)-1piperazineethanesulfonic acid (HEPES, Sigma-Aldrich) and 2.5 mM probenecid, which prevents the active transport of Fluo-4 out of the cells [36]. Cells were incubated for 40 min, and then mean fluorescence across each well was measured every 1.6 s for 90 s using a Flexstation 3 (Molecular Devices, Wokingham, UK) with excitation at 485 nm and emission at 525 nm. Test solutions (20 μL volume) were added after 16 s and included media-only control and a range of oxytocin standards (0.1 nM–0.01 mM in serum-free media) in each experiment.

To verify that oxytocin-induced [Ca^2+^]_i_ changes in our assay were mediated via oxytocin receptors and not vasopressin V_1A_ or V_2_ receptors (which oxytocin has lower affinity for [37]), we examined the impact of selective antagonists [37] for oxytocin (L-368,899; 0.1–10 μM, Tocris Bioscience, Bristol, UK), V_1A_ (SR 49059; 0.1 μM, Sigma-Aldrich) and V_2_ receptors (tolvaptan; 1 nM, Sigma-Aldrich). To assess the complexation of oxytocin with PLR (which should temporarily decrease the bioactivity of oxytocin in our assay), we selected an 0.1 μM concentration of oxytocin (which corresponds to the EC_50_ value in our reporter assay; Supplementary Figure S1) for examination with a range of PLR concentrations. To demonstrate the non-covalent nature of the oxytocin–PLR complex, we verified that the bioactivity of oxytocin could be restored by heparin competition (10 μg/mL).

#### 2.7.3. DLS and TEM

The hydrodynamic diameter, polydispersity index (PDI) and zeta potential of oxytocin–PLR nanocomplexes in ultrapure nuclease-free water were measured by DLS using a Malvern Panalytical Zetasizer (Malvern, UK) and ZS Xplorer software (v3; Malvern Panalytical Ltd). Morphology and particle size of oxytocin–PLR nanocomplexes were confirmed via TEM (Tecnai™ G2 12; FEI, Peabody, MA, USA) with an accelerating voltage of 100 kV. Briefly, a drop of sample (20 μL) was cast onto a 200-mesh copper grid with carbon film. Excess solution was removed, and grids were air-dried. They were stained with uranyl acetate stain and allowed to dry before imaging [6].

### 2.8. Effect of PLR on Oxytocin Transcytosis across an RPMI 2650 Cell Barrier

RPMI 2650 cells were seeded on polyester membrane inserts (12 mm diameter, 0.4 μm pore size; 3460; Corning, Deeside, UK) and transcytosis was assessed as described above (Section 2.5), with the exception that inserts were transferred to new wells with fresh basal media after each collection, and oxytocin concentrations in apical and basal fluid samples were determined using calcium fluorimetry (Section 2.7.2). We assessed molar oxytocin/PLR ratios that demonstrated complexation in our bioactivity reporter assay. However, absolute concentrations of both peptides applied to the apical chamber were increased by 12-fold to ensure that oxytocin levels in basal samples remained around the EC_50_ value in the reporter assay. 

### 2.9. Effect of Oxytocin–PLR Nanocomplexes on RPMI 2650 Cell Viability

Viability assays were performed alongside transcytosis measurements as described above (Section 2.5), with the exception that cells were incubated with the PrestoBlue™ reagent for 30 min and 100 μL of samples was analyzed using a SpectraMax M2e plate reader with SoftMap Pro 7.1.2 software (Molecular Devices). 

### 2.10. Statistical Analyses

All assays involved three technical replicates and were repeated on a minimum of three separate occasions to give n = 3–6 biological replicates. 

All analyses were performed using GraphPad Prism (v10.1.2). Normality was confirmed using Shapiro–Wilk’s test prior to use of parametric analyses, and in the case of insulin uptake, data log_10_ transformation was required to achieve this. Data were analyzed by one-way ANOVA (with media composition as the variable), two-way ANOVA (media composition × time) or two-way repeated measures ANOVA with Geisser–Greenhouse’s correction for unequal variance (media composition x time as a within-subjects factor in cases where samples were collected from the same cultures at different time points). ANOVAs were followed by Tukey’s or Sidak’s multiple-comparison post hoc tests. *p* < 0.05 was regarded as statistically significant, and data are presented as mean ± standard error of the mean (s.e.m.) with individual data points also shown on histograms.

## 3. Results and Discussion

### 3.1. PLR Increases Insulin Uptake and Transcytosis at Concentrations That Do Not Reduce RPMI 2650 Barrier Integrity or Cell Viability

#### 3.1.1. Insulin Uptake

**Figure 2:**
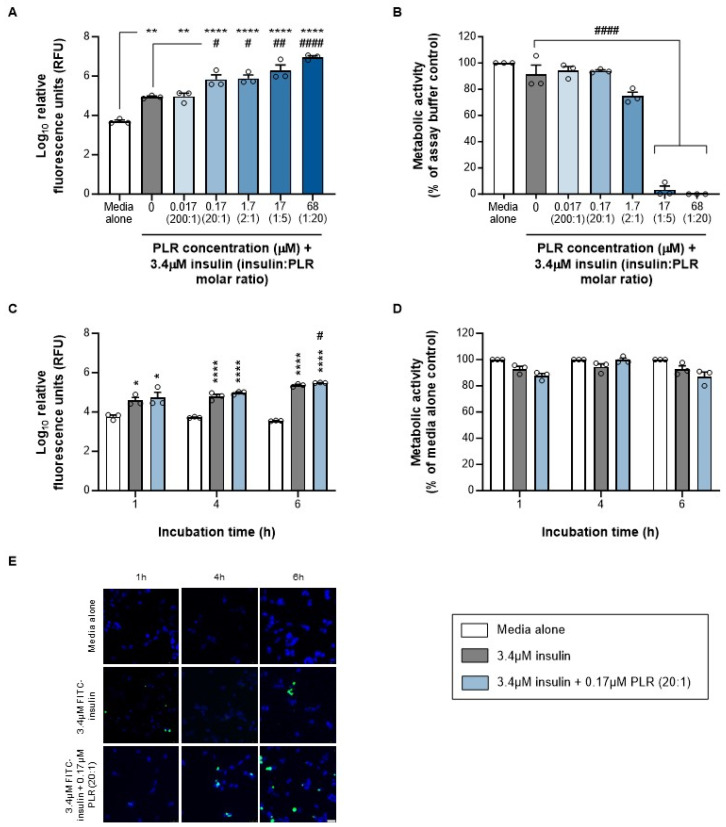
PLR increases insulin uptake into RPMI 2650 nasal epithelial cells at concentrations that do not reduce cell viability. Data are shown as mean ± s.e.m. (with individual data points also shown) for (**A**) insulin uptake and (**B**) cell viability following 24 h of incubation with insulin alone (gray bars) and increasing PLR concentrations (blue bars), (**C**) insulin uptake and (**D**) cell viability following 1–6 h of incubation with insulin alone (gray bars) or 20:1 molar ratio of insulin/PLR (blue bars), as well as (**E**) representative images showing cells that have taken up FITC-insulin (green) as proportion of total cells, labeled with DAPI (blue); scale bar = 25 μm. */**/**** *p* < 0.05/0.01/0.0001 versus media alone (control) and #/##/#### *p* < 0.05/0.01/0.0001 versus insulin alone (one-way ANOVA with Tukey’s multiple-comparison post hoc test or two-way ANOVA with Sidak’s multiple-comparison post hoc test). Viability data from (media alone) control (**B**,**D**) have zero variance and were excluded from statistical analyses.

Transport across epithelial barriers can involve paracellular routes through tight junction pores (<15 nm) or transcellular pathways that require uptake across apical and exocytosis from basal membranes [38]. Simple uptake is an essential prerequisite for transcellular delivery, so our first examination of the well-characterized insulin–PLR nanocomplex in nasal (as opposed to gastrointestinal [6]) epithelial cells focused on this process in liquid-covered cultures. We initially assessed our full range of insulin/PLR ratios following a 24 h incubation period and found that the uptake achieved by insulin alone was enhanced by four of the five combinations (20:1 and 2:1 by 10- to 11-fold, 1:5 by 35-fold and 1:20 by 106-fold; Figure 2A). However, the highest two, which involved 17 and 68 μM PLR, were associated with a 96–99% reduction in epithelial cell viability at this time point (Figure 2B), so they were excluded from further study. Flow cytometry and confocal microscopy assessments of the 20:1 insulin/PLR ratio over a 1–6 h period confirmed that enhanced uptake was also detectable at 6 h (Figure 2C, 1.3-fold and Figure 2E, 3.25-fold, respectively) without any impact on viability (Figure 2D). However, we acknowledge that the effect of the 20:1 combination at 6 h was relatively modest, and we therefore progressed both this and the 2:1 ratio (which involve 0.17 and 1.7 μM PLR) to transcytosis studies.

#### 3.1.2. Insulin Transcytosis

Transferring RPMI 2650 cells from liquid-covered culture to the air–liquid interface by the eighth day after seeding is recognized to stimulate physiologically relevant tight junction and drug transport protein expression [18,31]. Cells are routinely used for epithelial permeability studies on days 20–21 [18,19,20,21,22,23,31,32], and at this point, we were able to replicate TEER values suggestive of tight junction formation (Supplementary Figure S2a), which were within the typical range for these cultures [39] and excised human nasal mucosa (60–180 Ω/cm^2^ [18]). Cells maintained at the air–liquid interface impeded the passage of 4.4 kDa FITC-dextran (Supplementary Figure S2b), and their P_app_ values (1.78 ± 0.08 × 10^6^ cm/s) were also within reported ranges for these cultures (0.68 ± 0.03 to 2.48 ± 0.72 × 10^6^ cm/s [39]).

**Figure 3:**
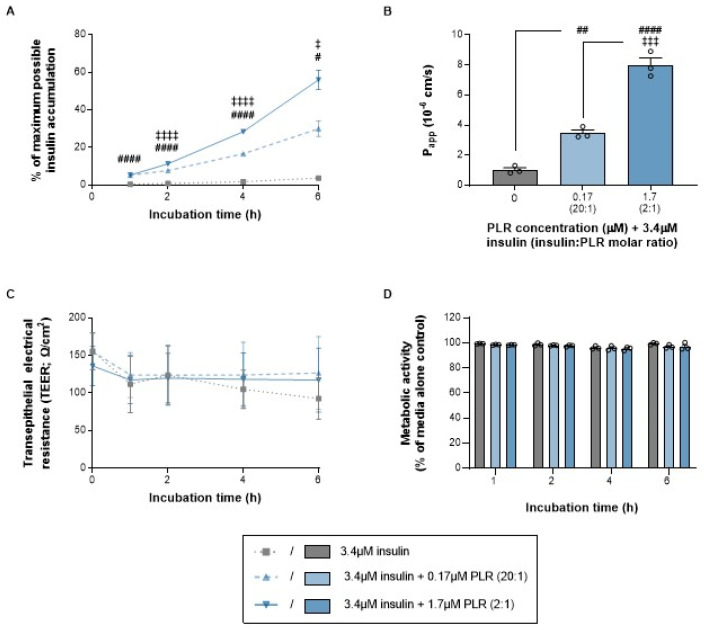
PLR increases insulin transcytosis across an RPMI 2650 nasal epithelial cell barrier (cultured on transwell inserts at the air–liquid interface) at concentrations that do not reduce transepithelial electrical resistance (TEER) or cell viability. The data are shown as the mean ± s.e.m. (with individual data points also shown on the histograms) for (**A**) the time course of insulin accumulation in the basal chamber; (**B**) apparent permeability (P_app_) across the 6 h period as a whole; and (**C**) TEER and (**D**) cell viability time courses following incubation with insulin alone (gray lines/bars) and increasing PLR concentrations (blue lines/bars). #/##/#### *p* < 0.05/0.01/0.0001 for both 20:1 and 2:1 molar ratios of insulin/PLR versus insulin alone and ^‡/‡‡‡^/^‡‡‡‡^ *p* < 0.05/0.001/0.0001 for a 2:1 molar ratio of insulin/PLR versus 20:1 (one-way ANOVA, two-way ANOVA or two-way repeated-measures ANOVA with Geisser–Greenhouse’s correction for unequal variance, all followed by Tukey’s multiple-comparison post hoc test).

The transcytosis of insulin alone across the RPMI 2650 barrier was minimal (Figure 3A,B), which mirrors the findings in Caco-2 gastrointestinal epithelium [6] and supports our rationale for seeking an improved delivery strategy. PLR produced a concentration-related increase in insulin accumulation within the basal chamber, which was evident from the first time point (1 h) onwards and resulted in an 8-fold elevation (20:1 insulin/PLR, which involves 0.17 μM PLR) or 15-fold elevation (2:1 insulin/PLR, which involves 1.7 μM PLR) compared to insulin alone by 6 h (Figure 3A). This translated to 3.4- and 7.8-fold elevations in P_app_ for the 6 h period as a whole (Figure 3B). Importantly this enhancement occurred without any decrease in the TEER (Figure 3C) or metabolic activity (Figure 3D) at any time point. We interpret that, as well as increasing insulin uptake (Section 3.1.1), our PLR peptide with its LK15 endosomal escaping sequence (to minimize intracellular entrapment) also achieved a selective increase in transcytosis across an intact nasal epithelial layer—rather than compromising barrier integrity. This is not the case for other approaches to improve insulin delivery. For example, chitosan and cyclodextrin derivative nanoparticles increase permeability to insulin by modulating tight junctions, which reduces the TEER [40]. The effect of PLR on insulin transcytosis appeared superior to that of lipid-based nanoemulsion strategies at equivalent time points [41], and it was broadly comparable to that of solid lipid and poly(lactic-co-glycolic acid) nanoparticles [42] in in vitro models of nasal delivery. Some of these approaches also improved insulin delivery from the nose to plasma in vivo [40,41], which shows that the in vitro assays used here have good predictive validity and suggests that similar in vivo efficacy may be realized with insulin–PLR in the future. Additional insulin formulations have been assessed in vitro using Calu-3 airway epithelium rather than nasal epithelium as in this study. For example, sperminated gelatin tight junction modulators [43], phenylboronic acid nanoparticles [44] and arginine-coated self-emulsifying nanoglobules [45] all enhance insulin uptake or transcytosis to improve insulin bioavailability or bioactivity during in vivo studies using rodents. Because we have previously shown that orally administered insulin–PLR normalizes blood glucose in diabetic mice [6], our working hypothesis is that our nanocomplexes dissociate during the transcytosis process to release biologically active intact insulin rather than inactive insulin fragments.

### 3.2. Oxytocin Complexes with PLR and Retains Its Biological Activity upon Dissociation 

#### 3.2.1. Fluorescence Quenching and Dequenching

**Figure 4:**
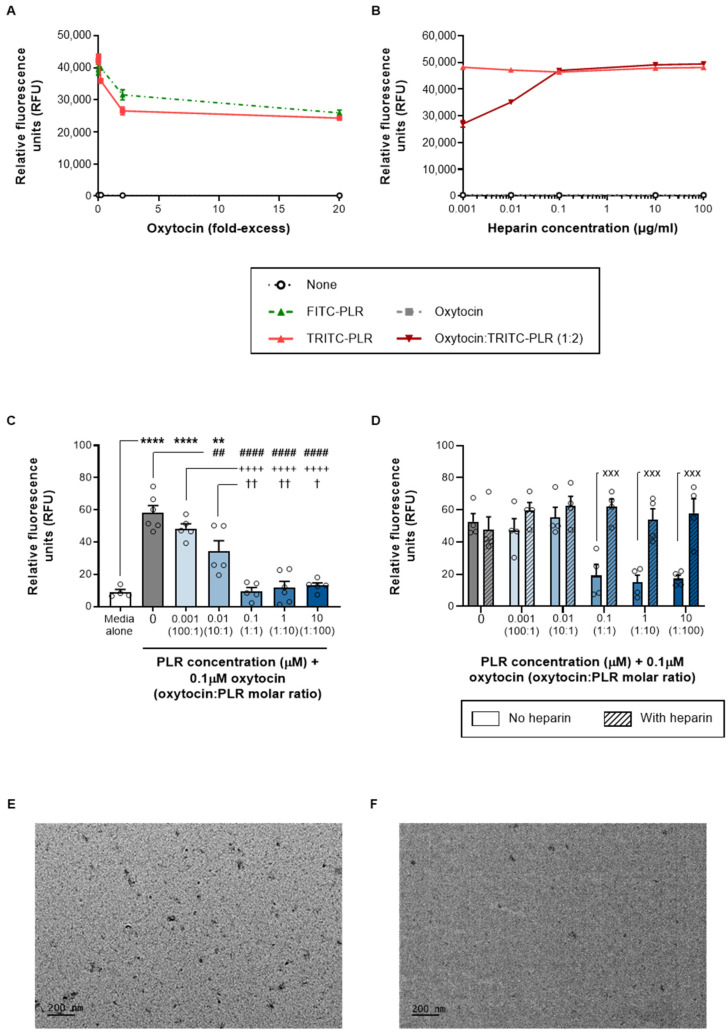
PLR complexes with oxytocin in a reversible, heparin-sensitive manner. The data are shown as the mean ± s.e.m. (with individual data points also shown on the histograms) for (**A**) the quenching of FITC-PLR (green dotted line) and TRITC-PLR (red solid line) fluorescence by increasing concentrations of oxytocin; (**B**) the dequenching of oxytocin/TRITC-PLR fluorescence by increasing concentrations of heparin (dark red line); as well as (**C**) the bioactivity of oxytocin alone (gray bars) in Hs 578T cells, which is reduced by increasing PLR concentrations (blue bars) and (**D**) restored by heparin (diagonal shaded bars). The images were obtained from a TEM examination of (**E**) 1:1 and (**F**) 1:10 ratios of oxytocin/PLR. **/**** *p* < 0.01/0.0001 versus media alone (control), ##/#### *p* < 0.01/0.0001 versus oxytocin alone, ++++ *p* < 0.001 versus a 100:1 molar ratio of oxytocin/PLR, †/†† *p* < 0.05/0.01 versus a 10:1 molar ratio of oxytocin/PLR, and xxx *p* < 0.001 versus the same oxytocin/PLR ratio in the absence of heparin (one-way ANOVA or two-way ANOVA with Tukey’s multiple-comparison post hoc test).

We previously developed a fluorescence-based assay for insulin–PLR complexation [6] and adopted a similar approach here for our first evaluation of oxytocin–PLR. Progressive fluorescence quenching of both FITC-PLR and TRITC-PLR was observed with increasing oxytocin concentrations and was equivalent to a 40% signal decrease with a 20-fold molar excess of oxytocin (Figure 4A). As reported for insulin–PLR [6], complexation occurred rapidly (≤15 min) upon simple mixing and was disrupted in a concentration-dependent manner by heparin (Figure 4B), which binds to the P21 domain of PLR and thus limits the scope for other peptide interactions. The readily reversible nature of the oxytocin–PLR interaction suggests against covalent bonds that would be likely to interfere with the binding of oxytocin to its receptors and adversely affect therapeutic activity.

#### 3.2.2. Bioactivity Studies in Hs 578T Cells

Unlike the sequence of insulin, that of oxytocin is not amenable to fluorescent labeling [25]. We therefore developed a calcium signaling reporter assay for use as an alternative readout to fluorescent peptide levels in media samples from transcytosis studies. However, the assay can also provide valuable insights into oxytocin–PLR complexation. Before embarking on these studies, we verified that oxytocin-induced increases in [Ca^2+^]_i_ in Hs 578T cells were mediated via the activation of Gα_q_-coupled oxytocin receptors and are thus sensitive to blockade with the selective oxytocin receptor antagonist L-368,899 (Supplementary Figure S1a). Oxytocin also has affinity for Gα_q_-coupled V_1A_ and V_2_ receptors (albeit 150- and >4000-fold lower than for oxytocin receptors [37]). But importantly, oxytocin-induced [Ca^2+^]_i_ increases in our assay appear independent of these receptors since they were not sensitive to the V_1A_ receptor antagonist SR 49059 or V_2_ receptor antagonist tolvaptan (Supplementary Figure S1b). These findings are consistent with reports that Hs 578T cells express oxytocin but not vasopressin receptors [26].

Pre-incubation with increasing concentrations of PLR reduced oxytocin-induced calcium transients. This effect reached significance with four of the five combinations and encompassed the same molar ratios where fluorescence quenching was observed (10:1 −41%, 1:1, 1:10 and 1:100 −78 to −84%; Figure 4C). Comparisons between oxytocin/PLR ratios confirmed that the 1:1, 1:10 and 1:100 mixtures had a greater impact on oxytocin-induced calcium transients than the 10:1 ratio. We interpret reduced biological responses to oxytocin in the presence of PLR as evidence for successful complexation, which would be expected to temporarily reduce free oxytocin availability and thus also oxytocin receptor activation. Crucially, heparin fully reversed the inhibitory effect of PLR, restoring oxytocin-induced calcium transients to levels seen with oxytocin alone (Figure 4D). This parallels findings from our dequenching assay and suggests a reversible interaction with PLR that should enable oxytocin dissociation for subsequent oxytocin receptor activation. Although the addition of heparin to decouple the complex could be seen as somewhat artificial, we predict similar competition in vivo from cell surface heparan sulfate proteoglycans [5]. 

PLR has a net positive charge and has been previously shown to complex with negatively charged nucleic acids [5] and peptides [6], but this represents the first suggestion that it can interact with peptides that have a weak positive charge. We propose that this may involve van der Waals interactions between hydrophobic side chains (on 56% of oxytocin residues and 32% of PLR residues), which preferentially associate with each other rather than aqueous media. On the basis of data from our complexation work, we progressed the 1:1 and 1:10 oxytocin/PLR ratios to transcytosis studies. We did not pursue the 1:100 ratio because scaling up absolute peptide concentrations in the apical chamber (to ensure the basal chamber oxytocin levels were optimal for the detection assay) would have required an apical PLR concentration (120 μM) above that shown to reduce RPMI 2650 viability (Figure 2B).

#### 3.2.3. DLS and TEM

A DLS examination of the 1:10 oxytocin/PLR ratio (which was found to enhance transcytosis; Section 3.3) revealed a hydrodynamic diameter of 194.4 ± 141.3 nm (range 65.6–388.7 nm; n = 5) and a zeta potential value of +0.14 ± 0.16 mV (n = 3). The TEM showed that the 1:1 and 1:10 oxytocin–PLR nanocomplexes (Figure 4E,F) had a similar appearance to those previously reported for insulin–PLR [6]. The average diameter shown by the TEM was 30.86 ± 9.6 nm for the 1:10 combination (Figure 4F).

### 3.3. PLR Increases Oxytocin Transcytosis at Concentrations That Do Not Reduce RPMI 2650 Barrier Integrity or Cell Viability

**Figure 5:**
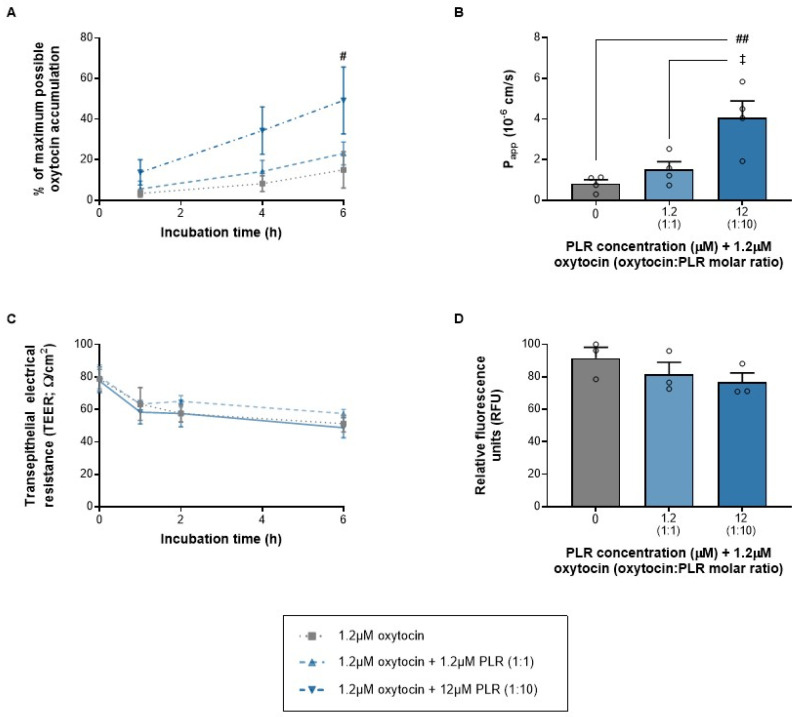
PLR increases oxytocin transcytosis across an RPMI 2650 nasal epithelial cell barrier (cultured on transwell inserts at the air–liquid interface) at concentrations that do not reduce the transepithelial electrical resistance (TEER) or cell viability. The data are shown as the mean ± s.e.m. (with individual data points also shown on the histograms) for (**A**) the time course of oxytocin accumulation in the basal chamber; (**B**) apparent permeability (P_app_) across the 6 h period as a whole; as well as (**C**) the TEER across the time course of transcytosis measurements and (**D**) cell viability at the end of the 6 h period following incubation with oxytocin alone (gray lines/bars) and increasing PLR concentrations (blue lines/bars). #/## *p* < 0.05/0.01 for both the 1:10 molar oxytocin/PLR ratio versus oxytocin alone and ^‡^ *p* < 0.05 for the 1:10 molar oxytocin/PLR ratio versus 1:1 (one-way ANOVA, two-way ANOVA or two-way repeated-measures ANOVA with Geisser–Greenhouse’s correction for unequal variance, all followed by Tukey’s multiple-comparison post hoc test).

Oxytocin is approximately 4.8 kDa smaller than insulin, but transcytosis across an RPMI 2650 barrier was still negligible (*p* > 0.05 versus insulin alone). The 1:10 oxytocin/PLR combination (which involved 12 μM PLR) was able to enhance oxytocin accumulation within the basal chamber at the 6 h time point, resulting in a 3.3-fold elevation compared to oxytocin alone (Figure 5A) and a 5-fold elevation in P_app_ for the 6 h period as a whole (Figure 5B). The minimum effective concentrations of PLR were higher for oxytocin than insulin, in both absolute and molar ratio terms, presumably due to a weaker interaction with oxytocin. But importantly, as with insulin–PLR, the elevation in oxytocin transcytosis occurred without any decrease in the TEER (Figure 5C) or metabolic activity (Figure 5D). The fact that oxytocin retained its bioactivity after transcytosis, without any need for heparin dissociation of the PLR nanocomplex, further supports our belief that PLR nanocomplexes indeed dissociate during transcytosis to release biologically active therapeutic cargoes. However, this will need to be assessed on a case-by-case basis for other PLR–peptide complexes.

Previous approaches to enhance the nose-to-brain delivery of oxytocin include phospholipid vesicles containing magnesium and the mucoadhesive polymer alginate (phospholipid magnesome [46]) and bovine serum albumin nanoparticles [47]. Both of these increased the behavioral effects of oxytocin in vivo, but to the best of our knowledge, neither formulation was examined in RPMI 2650 transcytosis assays beforehand, so direct comparisons with oxytocin–PLR data are not yet possible. 

## 4. Conclusions

This study is the first to demonstrate that our PLR delivery peptide improves the transcytosis of two contrasting therapeutic peptides, insulin and oxytocin, across an RPMI 2650 human nasal epithelial cell barrier cultured at the air–liquid interface. This in vitro assay is recognized to be particularly suitable for predicting nasal delivery [18,19,20,21,22,23], and although the cells do not differentiate to the same extent as three-dimensional MucilAir^TM^ cultures [48], comparative studies with an array of pharmaceutical agents suggest excellent correlation between these models [20]. We therefore advocate the continued evaluation of insulin–PLR and oxytocin–PLR formulations. Our ultimate goals are for PLR to improve the nose-to-plasma delivery of insulin to manage blood glucose levels [13,14], the nose-to-brain delivery of insulin for the treatment of Alzheimer’s disease, traumatic brain injury and cerebral ischemia [16], and/or the nose-to-brain delivery of oxytocin for the treatment of Autism Spectrum Disorders and schizophrenia [12]. Immediate next steps include conducting a more in-depth assessment of ciliotoxicity and cytotoxicity following repeated daily administration to nasal cultures [49], followed by progression to in vivo assessments in rodents, where factors such as mucociliary clearance, enzymatic degradation and mechanical shear stress are all additional barriers [50]. A combined investigation of insulin and oxytocin would also be of interest given that oxytocin may modulate glucose metabolism [51], while insulin appears capable of modulating oxytocin release [52].

## Data Availability

The raw data associated with the original contributions presented in this study are included in the supplementary material; further enquiries can be directed to the corresponding authors.

## Supplementary Materials

The following supporting information can be downloaded at https://www.mdpi.com/article/10.3390/pharmaceutics16101267/s1, Figure S1: Oxytocin-induced increases in [Ca^2+^]_i_ in Hs 578T cells are mediated by oxytocin receptors, not vasopressin V_1A_ or V_2_ receptors; Figure S2: RPMI 2650 cells cultured at the air–liquid interface (ALI) demonstrate higher barrier integrity than those under liquid-covered culture (LCC); Table S1: (a–t): Raw data used to produce our results shown in figures.
