# Supplementary material for: A Modified Cell-Penetrating Peptide Enhances Insulin and Oxytocin Delivery across an RPMI 2650 Nasal Epithelial Cell Barrier In Vitro
Source: Pharmaceutics. 2024 Sep 28;16(10):1267. doi: 10.3390/pharmaceutics16101267 (PMC11510563; doi:10.3390/pharmaceutics16101267)
Supplement: Supplementary file 1 [file pharmaceutics-16-01267-s001.zip › Supplementary Figure S1 and S2.pdf]

A

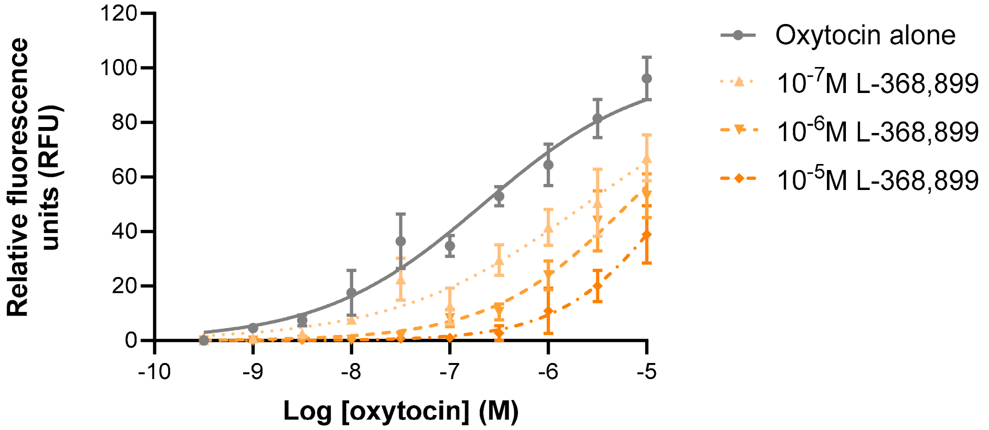

B

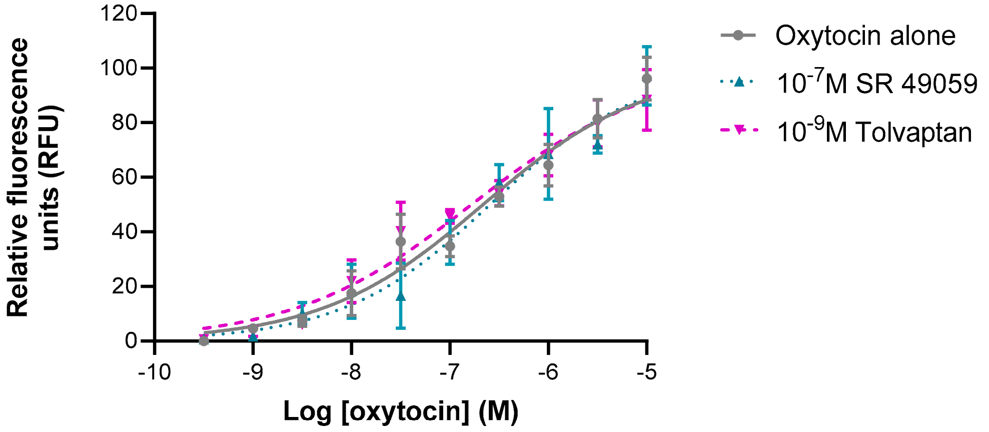

Figure S1. Oxytocin-induced increases in $[Ca^{2+}]_i$ in Hs 578T cells are mediated by oxytocin receptors, not vasopressin V_{1A} or V_2 receptors. Data are mean ± s.e.m. calcium fluorescence evoked by oxytocin alone (grey), which was (**a**) reduced by increasing concentrations of the oxytocin receptor antagonist L-368,899 (orange), as shown by a main effect of treatment on EC_{50} values ($F_{(3, 11)} = 445.6$, P<0.001) and significant difference (P<0.001) in the presence of all L-368,899 concentrations compared to oxytocin alone, but (**b**) not affected by the V_{1A} receptor antagonist SR 49059 (teal) or the V_2 receptor antagonist tolvaptan (magenta) as shown by a lack of main treatment effect ($F_{(2, 8)} = 1.01$, P=0.406; one-way ANOVA with Dunnett's post-hoc).

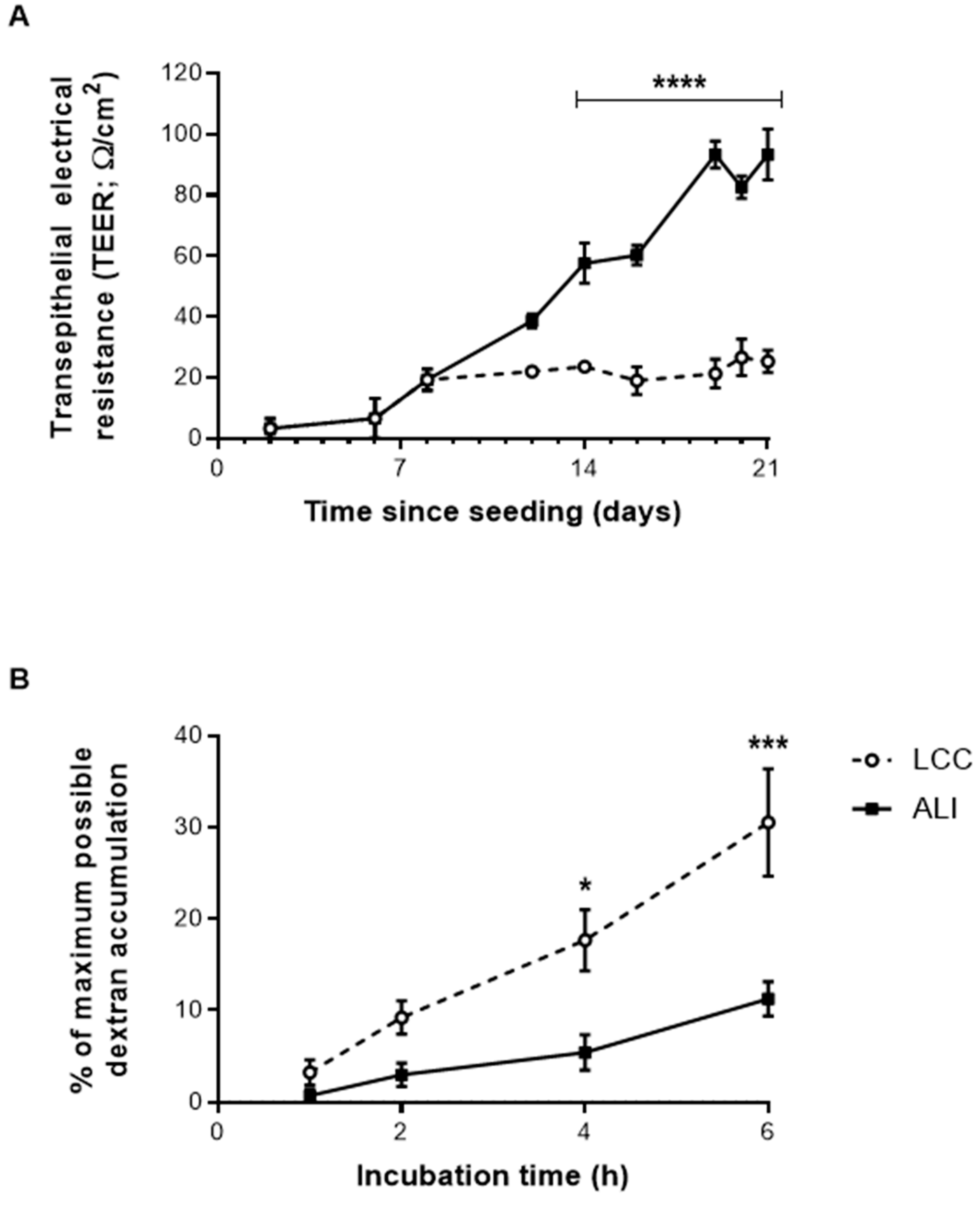

Figure S2. RPMI 2650 cells cultured at the air-liquid interface (ALI) demonstrate higher barrier integrity than those under liquid covered culture (LCC). Data are mean ± s.e.m. (**a**) transepithelial electrical resistance (TEER) and (**b**) dextran accumulation in the basal chamber, with cells cultured on trnaswell inserts at the ALI (solid lines) or under LCC (dashed lines). */***/**** $P<0.05/0.001/0.0001$ ALI versus LLC (two-way repeated-measures ANOVA with Sidak's multiple-comparison post-hoc).
